# Phenotypic plasticity underlies seasonal and latitudinal variation in thermal tolerance in a native bee

**DOI:** 10.1002/ecy.70183

**Published:** 2025-09-04

**Authors:** Matt C. Elmer, Keyne Monro, Harley Thompson, Aidan Stuckey, Vanessa Kellermann

**Affiliations:** ^1^ School of Biological Sciences Monash University Clayton Victoria Australia; ^2^ School of Agriculture Biomedicine and Environment La Trobe University Bundoora Victoria Australia

**Keywords:** acclimation, adaptation, cold tolerance, *Exoneura robusta*, heat tolerance, Hymenoptera, latitudinal cline, seasonal plasticity

## Abstract

Climate change threatens biodiversity and ecosystem services around the globe. Despite the importance of native bees as pollinators, there is evidence of global declines, and we know very little about how climate shapes their distributions now and into the future. In the current study, we combined large‐scale seasonal field sampling and experimental acclimation to examine whether populations of an Australian bee, *Exoneura robusta*, vary in their capacity to adapt to different climates. Collecting female bees across a latitudinal cline and examining heat and cold tolerance, we found populations did not vary in their heat tolerance along a latitudinal gradient. In contrast, bees from higher latitudes tended to be more cold‐tolerant than bees from lower latitudes, but the relationship between cold tolerance and latitude differed between summer and spring (post‐winter). Such seasonal variation suggests that phenotypic plasticity plays a role in shaping cold tolerance, as bees are likely to belong to the same generation from summer to spring. To untangle the roles of plasticity and genetic variation in shaping variation in thermal tolerance across seasons, we acclimated adult females from three populations spanning the species' distributional range to either 21 or 26°C in glasshouses (approximating summer and spring/autumn temperatures experienced throughout their range). We then estimated heat and cold tolerance. Contrasting acclimation responses observed in the glasshouses to those observed in the field point to phenotypic plasticity in cold tolerance rather than genetic variation underpinning population variation. In contrast, heat tolerance varied little in the field and in our glasshouse experiments. These results suggest bees may have little capacity to increase their heat tolerance, which is high at ~47°C, via genetic or plastic responses as climate changes.

## INTRODUCTION

Climate change threatens biodiversity and ecosystem functions across the globe (Bellard et al., 2012). Insects provide many of these functions but, as ectotherms, have a limited ability to thermoregulate, making climatic variables such as temperature particularly important in driving their distributions (Kellermann et al., 2009). As climates change, species may shift their distributions to track optimum climates or adapt in situ (Sgrò et al., 2016). Adapting to climate change can involve genetic change (shifts in allele frequencies across generations) or phenotypically plastic change (nongenetic shifts in phenotype across environments) in key traits linked to climate (Seebacher et al., 2015; Sgrò et al., 2016). While genetic change will likely underpin species adaptation to climate change in the long term, plastic responses may be critical for persistence in the short term (Gunderson & Stillman, 2015; Riddell et al., 2018; Sgrò et al., 2016). How vulnerable species are to climate change will therefore depend on the pace of climate change and the capacity of species to respond genetically and plastically both in the long and short term. However, divergent selection pressures across climates mean populations may vary in their genetic and plastic responses (Cicchino et al., 2023; Hoffmann et al., 2002; Tonione et al., 2020). Understanding the extent to which traits are underpinned by genetic and plastic variation is needed to understand the time scales at which populations can respond to climate change.

For species that can be reared in the laboratory, it is possible to use common garden experiments to untangle genetic and plastic effects on responses to climate (Hoffmann et al., 2002; Schou et al., 2015). Studies of insect species sampled across spatial gradients and reared in common environments have shown that populations often vary in traits that are likely important for adapting to climate change, including heat and cold tolerance (Hoffmann et al., 2002). These studies have shown heat and cold tolerance often vary in ways we would expect based on climatic adaptation. For example, populations of *Drosophila* show latitudinal clines for heat and cold tolerance, where heat tolerance is higher in warmer, low‐latitude environments and cold tolerance is higher in cooler, high‐latitude environments (Hoffmann et al., 2002; Ranga et al., 2017). Heat and cold tolerance are also phenotypically plastic in many insects (Marshall & Sinclair, 2012; Sgrò et al., 2016), such that species acclimated to hot or cold temperatures (during development or as adults) show increased heat or cold tolerance, respectively (van Heerwaarden et al., 2016). Studies have shown that plasticity in climate‐relevant traits, such as thermal tolerance, can vary among populations, but these studies are often limited to a handful of populations in laboratory‐based model species (Barley et al., 2021; Mathur & Schmidt, 2017; Sgrò et al., 2010; van Heerwaarden & Sgrò, 2011). Consequently, the extent to which plasticity contributes to current and future climate adaptation across populations remains poorly understood.

There are, however, limitations to laboratory approaches. Many ecologically important species cannot easily be reared in the laboratory, and patterns of adaptation in laboratory environments do not always predict adaptation in nature (Schiffer et al., 2013). For organisms that cannot be reared in the laboratory, the challenge becomes how to untangle genetic and environmental effects on phenotypes to understand the capacity for adaptation. An alternative (or complementary) approach for these species is to examine how climate‐relevant traits vary spatially and temporally in the field—specifically, by leveraging natural variation in temperature across latitudes or elevations, and from season to season (Huey & Buckley, 2022). Field clines then tell us how traits vary in space across populations, while seasonal studies tell us how rapidly populations can shift trait expression in response to short‐term changes in climate. Some insects, for example, can shift thermal tolerance traits on short time scales (Oliveira et al., 2021; Terblanche et al., 2006), and these shifts are often in the direction predicted by climate adaptation (e.g., greater cold tolerance in winter). However, studies capturing seasonal variation in traits remain rare. New insights into how climate‐related traits vary across populations, and the extent to which traits can change across seasons, are needed to better understand the capacity of species to shift traits in response to climate change.

Bees are one of the most ecologically important groups of insects; however, very few species can be easily reared in laboratory environments. Bees play a key role as pollinators in both natural and agricultural ecosystems (Garibaldi et al., 2013). Despite their importance, there is evidence of native bee declines, and little is known about how climate influences their distributions or the extent of genetic and plastic variation in key traits shaping those distributions (Potts et al., 2010; Zattara & Aizen, 2021). Most research on climatic adaptation in bees is confined to honeybees (*Apis mellifera*) and bumblebees (*Bombus* spp.), representing the few managed eusocial species. Research outside of these taxa has often focused on bees from the family Megachilidae, such as *Megachile* and *Osmia* species (Hayes & López‐Martínez, 2021; Melone et al., 2024), some of which are managed species for pollination, with comparatively less research on wild bees from other families. Studies on population variation in bee species are rare but have found that field populations from colder environments tend to be more cold‐tolerant, as seen in *Drosophila*, while heat tolerance is only weakly associated with temperature in contrast to clinal patterns in *Drosophila* (Barreiro et al., 2024; Hoffmann et al., 2002, 2003; Pimsler et al., 2020). Studies on phenotypic plasticity are even rarer. The handful of studies to look at plasticity in the form of adult acclimation (again, predominately in honeybees and bumblebees) have found limited plastic capacity in both heat and cold tolerance (Gonzalez et al., 2024; Oyen et al., 2018; Percival et al., 2020; Poore et al., 2025; Sepúlveda & Goulson, 2023). As such, it remains largely unknown how most bee species will respond to changing climates.

In the current study, we examine how key climate‐related traits, heat and cold tolerance, vary across space and time in a common Australian native bee, *Exoneura robusta*—a univoltine stem‐nesting species in which adults overwinter in their nests. Leveraging the latitudinal climatic gradient along the east coast of mainland Australia, we compared heat and cold tolerance among eight populations sampled throughout a latitudinal range of more than 10° (~2000 km) in both summer and spring (after they have overwintered). To understand the extent to which thermal tolerance shifts via genetic variation and plasticity, we performed a 24‐day adult acclimation experiment, using two temperature treatments and three populations spanning the northern, central, and southern latitudinal ranges of the species on mainland Australia. By comparing how thermal tolerance changes across populations and seasons, we can examine the extent to which these traits vary across space and over short time scales needed to respond to climate change.

## METHODS

### Study system

**FIGURE 1:**
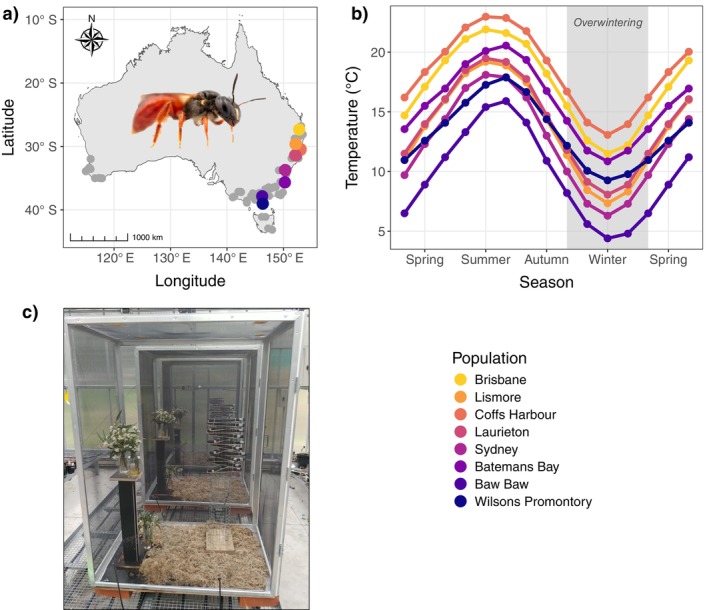
(a) Distribution of *Exoneura robusta* across Australia (gray points) and sampled populations (colored points), with an inset photo of *E. robusta* (photo credit: James B. Dorey). (b) Average monthly temperatures for each sampled population. We measured thermal tolerance of females in summer, and then again in early spring after bees had overwintered in their nests. (c) Bees from three populations spanning the latitudinal range of the species were then experimentally acclimated in separate cages for 24‐days, to gain insight into genetic and plastic contributions to thermal tolerance variation (photo credit: Matt C. Elmer).

*E. robusta* (Hymenoptera: Apidae) is an allodapine bee species native to Australia. It occurs along the east coast, from subtropical to temperate latitudes (25.7° S to 43.1° S; Figure 1a,b) and can be found in both montane and heathland habitats where it is an important pollinator (Bousjein et al., 2021). The species is univoltine, and varies in sociality, ranging from solitary nesting females to primitively eusocial colonies (Schwarz, 1986). These bees are <10 mm long (Walker, 2011) and are stem‐nesting. They commonly nest in fallen fronds of the tree fern *Alsophila australis* (syn. *Cyathea australis*) but can also be found in other plants with pithy stems such as lantana (*Lantana camara*) and blackberry (*Rubus fruticosus* aggregate). Egg laying commences around late winter/early spring as bees start to become active again after overwintering. Adults are then primarily active over spring and summer (September–February), as they progressively provision their brood, which mature into adults toward the end of summer. Colonies typically have a dominant female, which lays the majority of eggs, and subordinate females, which may lay eggs later, or disperse to found new colonies with other females where they will all lay eggs (Schwarz, 1986, 1987). Populations from warmer latitudes and heathland can produce two sets of brood; however, both sets of brood reach maturity and do not reproduce before overwintering and therefore belong to the same generation (Cronin & Schwarz, 1999a, 1999b). Bees overwinter in their nests (about April/May to August) before becoming active again the following spring (Schwarz, 1986). Across the distribution of *E. robusta*, populations typically experience summer and winter climates ranging from 26.8 to 0.6°C (mean monthly maximum and minimum temperatures; Fick & Hijmans, 2017).

### Latitudinal clines in thermal tolerance across seasons in the field

To determine whether or not bees varied in thermal tolerance across populations, we sampled bees from eight populations that encompassed the latitudinal range of *E. robusta* on the east coast of mainland Australia. Populations were sampled in summer (January and February 2022) and then resampled in early spring after overwintering (September 2022). Changes in trait clines across seasons are likely to reflect environmental effects on phenotypes, given bees collected over the summer and spring are likely to belong to the same generation in this univoltine species. For each of the eight populations, nests were collected from 1 to 6, nearby sites for a total of 24 unique collection sites (see Appendix S1: Table S1 for detailed collection site information). Collection sites ranged in elevation (from 30 to 1030 m above sea level [asl]) but were typically 800–1000 m asl. Bees were collected by identifying stems of typical nesting substrate containing nests (indicated by small circular holes at the ends of stems, characteristic of *Exoneura* spp.) (Schwarz, 1986). Nests were sealed with tape and transported to a field laboratory in insulated cooler boxes at ~10–14°C to prevent unnecessary thermal and nutritional stress and kept at these temperatures for one to two days before thermal tolerance assays. Nests were then opened, and bees were transferred into vials for thermal tolerance assays. Each nest contained 1–31 adult females, with a mean of 3–4, and bees from the same nest were divided between the two thermal tolerance assays as often as possible. The mean number of females assayed from a single nest was 2–3 for heat tolerance (range of 1–16), and 2–3 for cold tolerance (range of 1–15).

In summer, we examined 10–68 females per population to assess heat tolerance (with the exception of one population, represented by only a single individual), while in spring, this range was 37–68 females per population. For cold tolerance, we assessed 15–54 females per population in summer (with the exception of one population, represented by only two individuals), and 40–63 females per population in spring. A detailed breakdown of sample sizes for each thermal tolerance assay is provided in Appendix S1: Table S2.

### Thermal tolerance assays

To measure the heat and cold tolerance of bees, we estimated critical thermal maxima (CTmax) and minima (CTmin), respectively, using standard ramping methods (Chown et al., 2009). Bees were placed individually into 5‐mL airtight glass vials, then vials were randomly loaded into custom‐made racks and placed in a water bath at 26°C. Because we wanted to compare thermal tolerance across seasons, we kept the starting temperature the same, as it can influence estimates of critical thermal limits (Terblanche et al., 2007). The temperature of the water bath was then ramped at a rate of 0.1°C/min for CTmax, or cooled at the same rate for CTmin. We used this ramping rate as it is an ecologically relevant rate of temperature change in eastern Australia (Trewin, 2022), and it is commonly used for studying thermal tolerance in *Drosophila* (Overgaard et al., 2011; van Heerwaarden et al., 2016). We acknowledge that some hardening or thermal stress could have occurred. For each bee, CTmax and CTmin were defined as the temperatures at which all voluntary or involuntary movement ceased (Overgaard et al., 2011; van Heerwaarden et al., 2016). Due to technical limitations in the field when measuring the cold tolerance of Lismore bees in summer, we were unable to cool the temperature below −3.38°C. We therefore assigned this value to 25 bees with limited movement remaining as a conservative estimate of their cold tolerance. After assays, bees were stored individually in 100% undenatured ethanol for species identification via COI sequencing (see Appendix S1: Section S1 for details).

To determine if thermal tolerance temperatures relate to body size, we also measured wing length from 8 to 10 individuals per population. Wing length is linearly correlated with pupal mass (from which adults eclose) in *E. robusta* (Schwarz, Unpublished thesis) and therefore represents a reasonable proxy for body size in this species. Briefly, we removed and mounted bee forewings on glass slides, imaged wings under a Wild M3 dissector microscope (Leica, Heerbrugg, Switzerland) attached to a digital camera, then measured wing lengths from digital images in ImageJ (Schneider et al., 2012). To determine whether there was a relationship between body size and cold and heat tolerance, we looked for a correlation between average body size and average thermal tolerance in R (v4.3.1; R Core Team, 2024).

### Untangling genetic and plastic effects on thermal tolerance

To complement seasonal measures of thermal tolerance and gain insight into the capacity for bees to respond plastically to seasonal changes in temperature, we designed an adult acclimation experiment, where bees were kept in large cages (height, width, depth: 1500 × 820 × 1105 mm; Figure 1c) inside glasshouses and acclimated to two temperatures. The two temperature treatments were a warm treatment of 29:24°C diurnal cycle (mean = 26.4°C) and a cool treatment of 22:19°C diurnal cycle (mean = 20.6°C). The warm temperature treatment was chosen to reflect summer temperatures experienced along the latitudinal gradient, while the cooler temperatures reflected spring/autumn temperatures experienced along the latitudinal gradient. Both treatments were subject to natural lighting (~13 h light and 11 h dark) and temperatures were monitored with HOBO MX2201 temperature loggers inside cages.

For this experiment, we used three populations: Brisbane, Sydney, and Baw Baw (representing the northern range edge, mid‐latitudinal range, and southern range edge of *E. robusta* on mainland Australia, respectively). Nests from each population were collected and brought back to Monash University in late September 2022. For each population, bees from 15 nests were split evenly into two sets of 15 experimental nests, which went into either the warm or cool temperature treatments. The nests for each population were kept in separate cages such that there were three cages per treatment (one for each population) and six cages in total across the two temperature treatments. Nests were made of milled pine as outlined in Schwarz and Overholt (1993), and each experimental nest contained five females on average. We randomly divided bees between treatments with no distinction between dominant and subordinate females as this would require ovary dissections. Any eggs, larvae, or males present were also divided evenly between experimental nests to limit potential stress or behavioral change in females. Experimental nests containing bees were kept at ca. 4°C for four days prior to being set up in cages as this has been demonstrated to improve acceptance of the nest transfer (Schwarz & Overholt, 1993). Bees were provisioned with fresh flowers from native plants (particularly from the family Myrtaceae) every 2 to 3 days and were given fresh honey water (1:1 ratio) via saturated cellulose sponges daily. Each cage was also provided bee pollen in a 70‐mL specimen jar with holes; however, bees were never observed using them. To provide access to water and humidity, the bottom of each cage was lined with sphagnum moss watered by sprinklers for 30 s each morning. Each cage held 65–88 adult females. Bees were acclimated for 24 days (October 8, 2022 to November 1, 2022) before their heat and cold tolerance was assayed using the same methods as previously described (see Appendix S1: Table S3 for a detailed breakdown of sample sizes). Ramping assays used a starting temperature of 24°C, which was intermediate to the two acclimation temperatures.

### Statistical analyses

To evaluate field clines in heat and cold tolerance, we fitted separate linear mixed‐effects models in R (v4.3.1; R Core Team, 2024) using the packages lme4 (v1.1‐33; Bates et al., 2015) and lmerTest (v3.1‐3; Kuznetsova et al., 2017). Fixed effects in our models included latitude, elevation, season, and all possible interactions between them. We used latitude and elevation as proxies of climate, rather than individual climatic variables, because climate can change in complex ways with latitude, and multiple or unmeasured climatic variables can covary with latitude. We used the latitude and elevation of each of the 24 unique collection sites that were used to collect bees for the eight populations. Random intercepts were included for population and nest to account for nonindependence of bees from each of these sources. We excluded males because they were rare (~9% of brood; Schwarz, Unpublished thesis), not found in some populations, and were more abundant in spring than summer, meaning seasonal changes in sex ratio could have biased results (Appendix S1: Table S1). Because latitude and elevation were on different scales, they were each mean‐centered and divided by the SD for analysis, with predictions back‐transformed to the original scale for plotting. Model assumptions were evaluated using the package DHARMa (v0.4.6; Hartig, 2022) and custom R scripts, which detected no serious violations. To test the significance of fixed effects, we used Type III Wald F tests with Kenward‐Roger df in the package car (v3.1‐2; Fox & Weisberg, 2019). We calculated the variance explained by fixed effects (marginal *R* ^2^) and our full model with fixed and random effects (conditional *R* ^2^) using the MuMIn package (v1.47.5; Bartoń, 2023). Model predictions were calculated using the ggeffects package (v1.2.2; Lüdecke, 2018), and slopes were calculated using the package emmeans (v1.8.6; Lenth, 2023).

To assess how populations varied in heat and cold tolerance across seasons in the field, we made separate linear mixed‐effects models. Models included population, season, and the interaction between them as fixed effects. Random intercepts were included for nest and collection site in our heat tolerance model, and for nests only in our cold tolerance model, as collection site explained zero variance when included in the model. For models that had significant effects of season and/or the population × season interaction, we used post hoc analyses to test if shifts in thermal tolerance from summer to spring were significant for each population. Similarly, for models with a significant effect of population, we used pairwise post hoc comparisons to test for population differences within each season. We did this using the package emmeans (v1.8.6; Lenth, 2023), and multiple testing was accounted for using the False Discovery Rate (FDR) method.

We modeled the experimental acclimation data with linear mixed‐effects models to determine whether populations acclimated to the two temperature treatments and whether this response varied across populations. Population (Brisbane, Sydney, Baw Baw), temperature (26 vs. 21°C), and the interaction between population and temperature were treated as fixed effects, while random intercepts were included for nests. We did not include collection site as a random effect because nests were collected from a single site for each acclimated population. Because we were interested in whether the acclimation response in the acclimation experiment reflected the seasonal response observed in the field, we analyzed females from the same three populations measured in the field using the modeling framework described above. For models with significant effects or interactions between population and season (or acclimation temperature), we used post hoc analyses to test if the shift in thermal tolerance across seasons (or temperature treatments) was significant for each population and to compare populations to each other within each season (or temperature treatment). For models with only a main effect of population, the thermal tolerance of populations was compared to one another in post hoc comparisons. We accounted for multiple testing with the FDR method. All values in the Results are presented as mean ± SE, unless otherwise specified.

## RESULTS

### Latitudinal clines in thermal tolerance across seasons in the field

**FIGURE 2:**
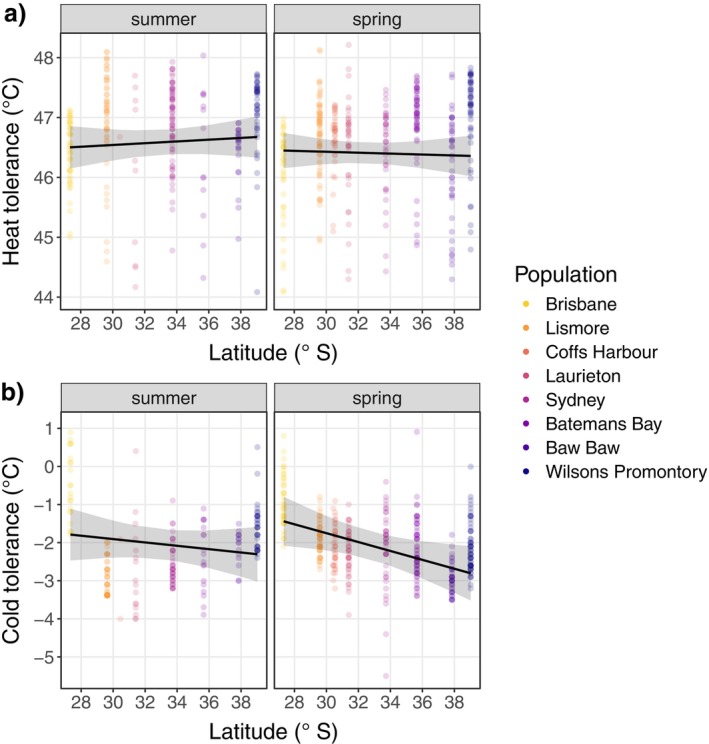
Latitudinal clines in thermal tolerance measured in the field during summer and early spring (post overwintering), for (a) heat tolerance and (b) cold tolerance in adult females. Trend lines show the fixed effect of latitude averaged over levels of elevation, with shading indicating the 95% CI.

Heat tolerance varied minimally across populations. In summer, the least tolerant population, Laurieton, averaged 46.01 ± 0.4°C, while the most tolerant population, Wilsons Promontory, averaged 47.09 ± 0.1°C (a range of 1.08°C). In spring, the least tolerant population, Brisbane, averaged 46.03 ± 0.12°C, and the most tolerant population, Wilsons Promontory, averaged 47.04 ± 0.10°C (a range of 1.01°C). Heat tolerance depended on the interaction between latitude and elevation, and on the interaction between elevation and season (Figure 2a; Table 1; see Appendix S1: Figure S1a for interaction plots). Consequently, the relationship between latitude and heat tolerance varied with elevation, and the strength of the effect of elevation differed between summer and spring. Across seasons, heat tolerance tended to be lower in the coldest environments (high latitude at high elevation) and the warmest environments (low latitude at low elevation, including Brisbane, around the warm range margin of *E. robusta*; Appendix S1: Figure S1a). In contrast, heat tolerance in both seasons tended to be highest in environments with more intermediate climates (high latitudes at low elevation, and low latitudes at high elevation; Appendix S1: Figure S1a). However, the effects of latitude, elevation, and season on heat tolerance were small, with only 11% of the variation in heat tolerance explained by these fixed effects, and the slope relating heat tolerance to latitude was weak regardless of season, suggesting very little local adaptation to climate (0.01 ± 0.02°C/degree south in summer, and −0.01 ± 0.02°C/degree south in spring, averaged over levels of elevation; Figure 2a, Table 1).

**TABLE 1:** Regression results for seasonal field clines in thermal tolerance.

Source of variation	Heat tolerance^a^				Cold tolerance^b^			
	*F*	df	*p*	σ^2^	*F*	df	*p*	σ^2^
Fixed effects								
Season	3.44	1, 205.8	0.065		0.88	1, 203.95	0.351	
Latitude	0.03	1, 3.64	0.879		2.64	1, 4.58	0.171	
Elevation	0.07	1, 5.56	0.805		2.14	1, 9.66	0.175	
Season × Latitude	1.04	1, 205.74	0.31		11.39	1, 196.42	**0.001**	
Season × Elevation	5.34	1, 167.68	**0.022**		16.68	1, 158.82	**<0.001**	
Latitude × Elevation	7.37	1, 4.49	**0.047**		0.02	1, 8.46	0.896	
Season × Latitude × Elevation	0	1, 217.76	0.95		12.27	1, 217.72	**0.001**	
Random effects								
Nest intercept				0.12				0.05
Population intercept				0.03				0.23

*Note*: Bold text indicates significance at *α* = 0.05.

^a^ Marginal *R* ^2^ = 0.11; Conditional *R* ^2^ = 0.34.

^b^ Marginal *R* ^2^ = 0.18; Conditional *R* ^2^ = 0.51.

Cold tolerance varied more than heat tolerance across populations and seasons. In summer, the least cold‐tolerant population, Brisbane, averaged −0.49 ± 0.18°C and the most tolerant population, Lismore, averaged −3.00 ± 0.06°C (a range of 2.51°C). In spring, the least tolerant population, Brisbane, averaged −0.91 ± 0.11°C while the most tolerant, Baw Baw, averaged −2.93 ± 0.06°C (a range of 2.02°C). Cold tolerance was found to depend on a significant three‐way interaction between latitude, elevation, and season (Figure 2b; Table 1; see Appendix S1: Figure S1b for interaction plots), meaning the relationship between cold tolerance and latitude depended on elevation, and the shape of this interaction differed between summer and spring. In summer, cold tolerance varied more across elevation than latitude, with high‐elevation populations being more cold‐tolerant. In spring, however, cold tolerance varied more across latitude than elevation, with greater cold tolerance in higher latitudes, which generally experience cooler climates (Figure 2b; Table 1; see Appendix S1: Figure S1b). Correspondingly, the latitudinal cline in cold tolerance was weaker in summer (−0.04 ± 0.05°C/degree south), than in spring (−0.12 ± 0.05°C/degree south), averaged over levels of elevation (Figure 2b). The effects of latitude, elevation, and season explained 18% of the variation in cold tolerance (Table 1). We found no significant correlation between either heat or cold tolerance and body size (measured by wing length; Appendix S1: Figure S2).

### Changes in thermal tolerance across seasons: Field acclimation

**FIGURE 3:**
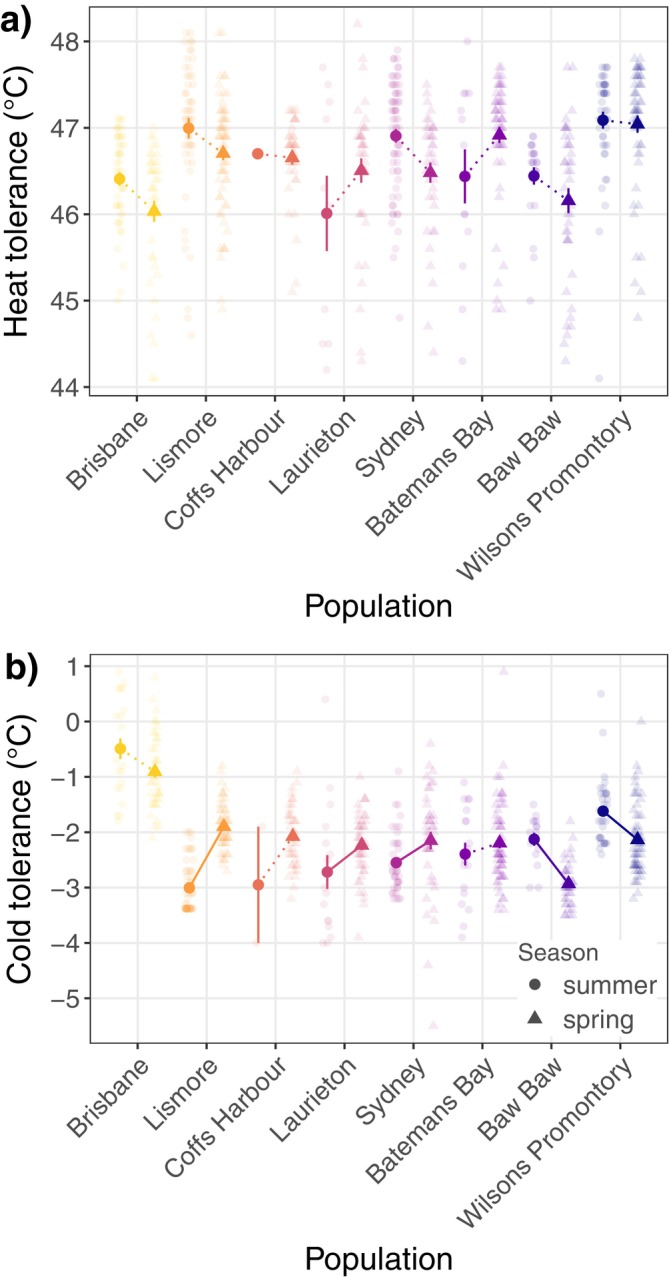
Field acclimation across seasons for each population in (a) heat tolerance and (b) cold tolerance in adult females. Solid points with error bars represent means and SEs. Solid lines indicate significant changes in tolerance across seasons, while dotted lines indicate no significant difference.

Based on seasonal shifts in temperature, we expected heat tolerance to decrease from summer to spring and cold tolerance to increase. For heat tolerance, we found six of the eight populations decreased their heat tolerance from summer to spring, as expected, with differences ranging from −0.05 to −0.43°C, although we found no significant effect of season or population × season interaction, suggesting there was no evidence for seasonal plasticity or population variation in plasticity (Figure 3a; Table 2).

**TABLE 2:** Regression results for field acclimation in thermal tolerance across populations and seasons.

	Heat tolerance^a^				Cold tolerance^b^			
	*F*	df	*p*	σ^2^	*F*	df	*p*	σ^2^
Fixed effects								
Population	3.21	7, 4.52	0.122		42.7	7, 199.47	**<0.001**	
Season	0.7	1, 100.38	0.404		4.07	1, 242.51	**0.045**	
Population × Season	1.67	7, 63.05	0.134		15.76	7, 199.47	**<0.001**	
Random effects								
Nest intercept				0.10				0.02
Site intercept				0.04				…

*Note*: Bold text indicates significance at *α* = 0.05.

^a^ Marginal *R* ^2^ = 0.15; Conditional *R* ^2^ = 0.35.

^b^ Marginal *R* ^2^ = 0.45; Conditional *R* ^2^ = 0.48.

Seasonal plasticity in cold tolerance varied in direction and magnitude across populations, as we found a significant population × season interaction (i.e., significant genotype × environment interaction; Figure 3b; Table 2). Three populations responded as expected, exhibiting increased cold tolerance from summer to spring, with differences ranging from −0.39 to −0.81°C, and two of these shifts were significant (in the two populations at highest latitude; Figure 3b; Table 2; Appendix S1: Table S4 for post hoc comparisons). Five populations decreased their cold tolerance, with differences ranging from 0.18 to 1.11°C, and three of these shifts were significant. Variance (σ^2^) in cold tolerance was similar across populations (σ^2^ = 0.48) and between seasons (σ^2^ = 0.47).

### Untangling genetic and plastic effects on thermal tolerance: Experimental acclimation

**FIGURE 4:**
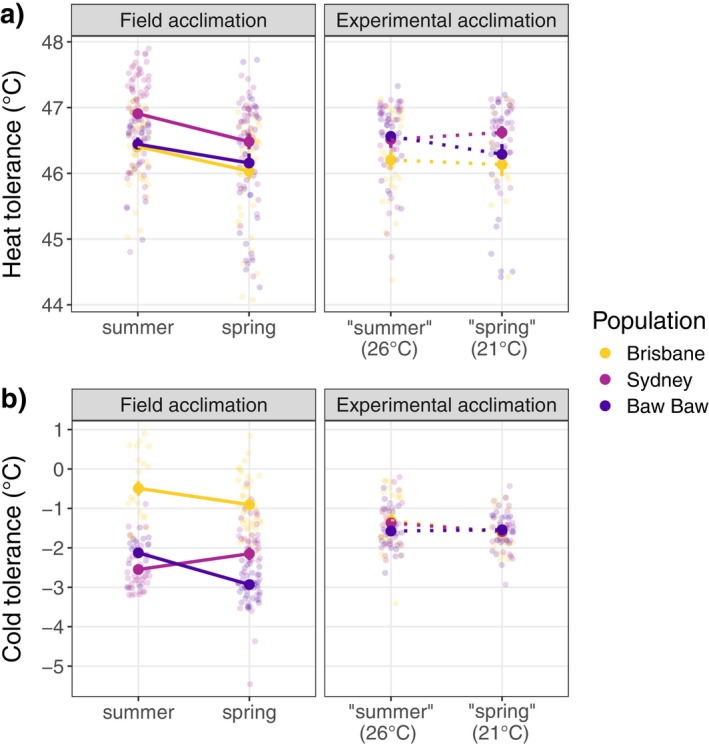
Comparison of field and experimental acclimation for (a) heat tolerance and (b) cold tolerance in adult females. Solid points with error bars represent means and SEs. Solid lines represent significant changes in tolerance across seasons or acclimation temperature treatment, while dotted lines indicate no significant difference.

We found no significant effect of acclimation, as heat tolerance did not differ between bees acclimated at 26°C and those acclimated at 21°C. However, Brisbane bees were always less heat tolerant than Sydney bees, suggesting that differences in heat tolerance across populations may have a genetic basis (Figure 4a; Table 3; Appendix S1: Tables S5 and S6 for post hoc comparisons).

**TABLE 3:** Field acclimation and experimental acclimation regression results for adult female *Exoneura robusta* from the three populations used in the 24‐day acclimation experiment.

	Heat tolerance				Cold tolerance			
	*F*	df	*p*	σ^2^	*F*	df	*p*	σ^2^
Field acclimation^a^								
Fixed effects								
Population	5.91	2, 97.3	**0.004**		110.32	2, 50.15	**<0.001**	
Season	12.66	1, 96.8	**0.001**		6.24	1, 58.3	**0.015**	
Population × Season	0.45	2, 97.3	0.636		11.65	2, 50.15	**<0.001**	
Random effects								
Nest intercept				0.15				0
Experimental acclimation^b^								
Fixed effects								
Population	3.23	2, 68.1	**0.046**		0.39	2, 59.15	0.682	
Acclimation temp.	0.34	1, 68.99	0.561		2.86	1, 60.23	0.096	
Population × Acclimation temp.	0.79	2, 68.1	0.458		1.13	2, 59.15	0.330	
Random effects								
Nest intercept				0.07				<0.01

*Note*: Bold text indicates significance at *α* = 0.05.

^a^ Heat tolerance: Marginal *R* ^2^ = 0.16; Conditional *R* ^2^ = 0.42. Cold tolerance: Marginal *R* ^2^ = 0.60; Conditional *R* ^2^ = 0.60.

^b^ Heat tolerance: Marginal *R* ^2^ = 0.06; Conditional *R* ^2^ = 0.22. Cold tolerance: Marginal *R* ^2^ = 0.04; Conditional *R* ^2^ = 0.05.

Similar to heat tolerance, we also found bees acclimated at 26°C (summer conditions) and those acclimated at 21°C (spring conditions) did not differ in their cold tolerance (Figure 4b; Table 3b). Unlike field comparisons, where the same three populations differed considerably in cold tolerance across seasons (Figure 4b, Table 3; Appendix S1: Table S7 for post hoc comparisons), populations converged on similar cold tolerance, following acclimation, suggesting that differences observed in the field are not underpinned by genetic variation and are instead, due to population variation in plasticity.

## DISCUSSION

Understanding species vulnerability to climate change requires knowing the extent to which species can shift traits both in the long and short term. Most studies on climatic adaptation in insects have focused on laboratory models, and therefore, our broader understanding of climatic adaptation in insects is still lacking. In the current study, we examined how climate shaped the distribution of an Australian bee, *E. robusta*, by determining whether thermal tolerance varied across populations collected along a latitudinal gradient. Averaged over levels of elevation, we found that cold tolerance varied latitudinally, whereas heat tolerance tended to show less latitudinal variation. Thermal tolerance also varied across seasons, although the extent of seasonal shifts differed among populations. A further experiment to untangle genetic and plastic contributions to thermal tolerance found no evidence of acclimation to experimental temperatures, but cold tolerance converged in all three populations, suggesting seasonal shifts in the field were likely driven by population variation in plasticity rather than genetic adaptation. In contrast, we saw no effect of acclimation for heat tolerance, but population differences remained, possibly due to genetic differences between them.

In the field, cold tolerance in *E. robusta* increased toward more temperate higher latitudes and higher elevations irrespective of season. These results are consistent with studies on insects in the field (bumblebees) (Pimsler et al., 2020) and in common garden conditions (*Drosophila* spp.) (Hoffmann et al., 2002). In contrast, heat tolerance tended to vary less across elevational and latitudinal gradients. While significant interactions between latitude and elevation, and elevation and season were found, they only explained a small proportion of the overall variation in heat tolerance (marginal *R* ^2^ = 0.11). Studies tend to find heat tolerance varies less across populations than cold tolerance (Addo‐Bediako et al., 2000; Kellermann et al., 2012). However, heat tolerance, in general, was high across the entire range of *E. robusta* (overall mean of 46.7 ± 0.03°C) contrasting other insects like *Drosophila* ~38.3°C (Kellermann et al., 2012), but consistent with other bee species (Gonzalez et al., 2024). It is therefore unlikely that absolute heat tolerance will limit the northern distribution of this species. However, the most northern population, Brisbane, was one of the least heat tolerant populations, which could suggest the warm range margin is determined by sublethal effects of temperature (Mokkapati et al., 2024; van Heerwaarden & Sgrò, 2021). Additionally, previous research suggests this species will only lay eggs post‐winter, even in the warmer populations (Cronin & Schwarz, 2001). The cost of overwintering in warmer environments could play a role in shaping the warm range edge, or dispersal capacity (Willi & Van Buskirk, 2019), or biotic interactions such as competition, predation, or availability of host plants could also be important (Paquette & Hargreaves, 2021). Additionally, thermal tolerance of gametes and earlier life stages such as embryos, larvae, and pupae may differ from adults and play a role in determining the warm range margin (Gudowska & Moroń, 2024; Pottier et al., 2022). It would be valuable to examine the thermal tolerance of these earlier life stages in future research.

Collecting bees across summer and spring (post‐overwintering), we found that populations differed in their capacity to shift their thermal tolerances across seasons. No populations showed a seasonal shift in heat tolerance, while five of the eight populations showed a seasonal shift in cold tolerance. However, the changes in cold tolerance were not always in the direction we would expect. Based on changes in temperature across seasons, if phenotypic plasticity was adaptive, we would expect greater cold tolerance in spring (when temperatures are lower) and reduced cold tolerance in summer (when temperatures are higher). While our results may reflect differences in plasticity across populations (akin to genotype × environment interactions), they may also be due to negative carryover effects (Kellermann & Sgrò, 2018; Schiffer et al., 2013), unmeasured trade‐offs between traits (van Heerwaarden & Kellermann, 2020), or the possibility that climatic shifts across seasons differed for different populations. Additionally, it could suggest that other factors, such as diet and food availability, may be driving population differences in seasonal plasticity (Kutz et al., 2019). These results also highlight the value of measuring traits in ecologically relevant contexts in the field, as controlled laboratory studies would likely have missed these unexpected effects.

Although populations varied in their acclimation response across seasons in the field, we found little evidence of an acclimation response in heat or cold tolerance in the laboratory (21 vs. 26°C acclimation treatment). The lack of acclimation in either heat or cold tolerance could be due to the climatic conditions in our experiment. Across treatments, the coolest mean nightly temperature was 19°C and the warmest mean daily temperature was 29°C. It may be that more extreme hot or cold temperatures are required to induce phenotypic plasticity in thermal tolerance in *E. robusta* (van Heerwaarden et al., 2024). Temperature thresholds for plasticity in thermal tolerance have been observed in tsetse flies (*Glossina pallidipes*), which have relatively low cold tolerance (CTmin roughly between 5 and 6.5°C). Cold tolerance plasticity was only detectable when flies were acclimated to 19°C or lower (Terblanche et al., 2006). Similarly, in *Drosophila*, thresholds have been linked to the induction of plastic responses in tolerance traits (van Heerwaarden et al., 2024), and only certain thermal treatments induced plastic responses in cold tolerance in *D. melanogaster* (Rako & Hoffmann, 2006). Given that *E. robusta* is more cold‐tolerant than both tsetse flies and *Drosophila*, it is possible that colder temperatures are needed to induce plasticity in cold tolerance. Follow‐up studies could evaluate if more extreme acclimation temperatures or longer durations induce plasticity in thermal tolerance in *E. robusta*.

While we found no acclimation response to our experimental temperature treatments, we found that the population differences in cold tolerance that we observed in the field all but disappeared. Similarly, for heat tolerance, population differences also disappeared, except between the populations of Brisbane and Sydney. An absence of population variation following acclimation suggests plastic rather than genetic effects likely drive the divergence observed in the field. Some studies in *Drosophila* have also found plasticity to have a greater contribution to variation in cold tolerance than genetic differences (Ayrinhac et al., 2004). Evidence for phenotypic plasticity by acclimation has rarely been observed in bees for either cold tolerance (Oyen et al., 2021; Sanchez‐Echeverria et al., 2019) or heat tolerance (Gonzalez et al., 2024). Research has typically found no acclimation capacity in thermal tolerance traits across honeybees (Percival et al., 2020), bumblebees (Oyen et al., 2018; Poore et al., 2025; Sepúlveda & Goulson, 2023) and wild bee species (Gonzalez et al., 2024). Studies on cold tolerance acclimation and the acclimation capacity of wild bee species are still scarce and should continue to be investigated in future research. Additionally, future research could further examine the influence of nesting biology on thermal tolerance and acclimation capacity, as stem‐nesting bees (such as *E. robusta*) are more likely to experience temperature fluctuations associated with the day, season, and changing climates compared with ground‐nesting species, which are likely more insulated from these changes (Campbell & Norman, 1998).

Estimating traits on field‐caught individuals means that environmental effects on phenotypes will increase trait variation, which could strengthen/inflate observed relationships between climate and traits if adaptive phenotypic plasticity (defined here as a change in phenotype that increases fitness in response to the environment) is present. Alternatively, we could see a weaker latitudinal association because of negative carryover effects on fitness (Schiffer et al., 2013). Furthermore, it is also possible that observed differences in thermal tolerance across seasons are underpinned by selection. Although we expect bees to belong to the same cohort across seasons, selection during overwintering could have selected more cold‐tolerant individuals. Similarly, the difference in thermal tolerance across seasons may result from drift or any overwintering mortality. Shifts in traits across seasons due to selection may be possible as experimental evolution studies in *Drosophila* have found rapid adaptive tracking of thermal tolerance across seasons (Rudman et al., 2022). Regardless, by comparing field clines across seasons, we can gain a better understanding of how the environment shapes trait variation.

## CONCLUSIONS

We found evidence for latitudinal and seasonal variation in cold tolerance, but variation across populations for heat tolerance was small, with no clear clinal patterns across latitude or season. The observed field variation in cold tolerance was likely underpinned by population variation in plasticity, given that we found populations converged in their cold tolerance in the lab acclimation experiment. While the estimates of heat tolerance were overall high, there was little evidence of plasticity. Persistent differences between populations in the acclimation experiment may indicate genetic variation underpinning heat tolerance. High estimates of heat tolerance suggest this species is not immediately vulnerable to climate change. However, little evidence of local adaptation, coupled with a northern distribution that is intrinsically linked to heat, could suggest that other sublethal effects of temperature, not measured here, may set the northern range limits of this species. Future research investigating levels of gene flow between populations could also provide key insight into the role of gene flow in contributing to variation in thermal tolerance. For instance, high migration rates may swamp and outweigh selection for emergent alleles with greater heat tolerance, preventing further northward expansion.

## CONFLICT OF INTEREST STATEMENT

The authors declare no conflicts of interest.

## Supporting information


Appendix S1:

## Data Availability

Data and code (Elmer et al., 2025) are available in Zenodo at https://doi.org/10.5281/zenodo.15845400.
