# Supplementary material for: Phenotypic plasticity underlies seasonal and latitudinal variation in thermal tolerance in a native bee
Source: Ecology. 2025 Sep 4;106(9):e70183. doi: 10.1002/ecy.70183 (PMC12411691; doi:10.1002/ecy.70183)
Supplement: Supplementary file 1 — Appendix S1: [file ECY-106-e70183-s001.pdf]

# Phenotypic plasticity underlies seasonal and latitudinal variation in thermal tolerance in a native bee

Matt C. Elmer, Keyne Monro, Harley Thompson, Aidan Stuckey, Vanessa Kellermann

*Ecology*

# Appendix S1

**Section S1: Species identification by COI sequencing**

Since female *Exoneura* are difficult to identify from morphology, we sequenced the mitochondrial barcoding gene, cytochrome c oxidase subunit 1 (COI), to confirm species identity. It was unnecessary when males were present in nests, since male *E. robusta* are easily identified from morphological characteristics including long, dense black hairs particularly on the face, and a similar face mark to females (Walker 2011). For sequencing, individual bees were chosen from a sample of nests per population, totalling 96/341 (28%) total nests collected. DNA was extracted from a single hind leg using 5% chelex solution (Walsh, Metzger, and Higuchi 1991), and the target region of COI was amplified using the primers LCO1490 (forward) and HCO2198 (reverse) (Folmer et al. 1994). PCR products were then purified and Sanger sequenced by Macrogen, Inc. (South Korea).

After Sanger sequencing by Macrogen, returned .ab1 files were inspected and cleaned in MEGA-X (v10.2.4, (Kumar et al. 2018)). Ambiguous base calls were removed (typically from the ends of reads as call quality declines), and FASTA files were created. We then downloaded FASTA files of COI sequences from identified *Exoneura* species from the National Center for Biotechnology Information (NCBI) (Sayers et al. 2021) and the Barcode of Life Data System (BOLD) (Ratnasingham and Hebert 2007) in R, using their API interfaces and custom R scripts. Reference COI sequences were also downloaded for *Brevineura xanthocypeata* to be used as an outgroup in phylogeny generation (Bousjein, Gardner, and Schwarz 2021). FASTA files of our samples, and reference sequences were combined, and a multiple sequence alignment was generated using MAFFT (v7.505 (2022/Apr/10 (Katoh and Standley 2013)). This alignment file was then used to generate a neighbour-joining tree in MEGA. Clades were then inspected for species membership, and the species of each sample was identified.

All sequences were *E. robusta* (92/96), with the exception of three sequences, which were unusable and one sequence, which belonged to a social parasite (*Inquilina* sp.), which could easily be identified by morphological characteristics, such as a concave face without a face mark (Smith & Schwarz, 2009). Individuals that were not *E. robusta* were removed from downstream analyses.

**References**

Bousjein, Nahid Shokri, Michael G. Gardner, and Michael P. Schwarz. 2021. "Demographic Stability of the Australian Temperate Exoneurine Bees (Hymenoptera: Apidae) through the Last Glacial Maximum." *Austral Entomology* 60 (3): 549–59. https://doi.org/10.1111/aen.12539.

Folmer, O., M. Black, W. Hoeh, R. Lutz, and R. Vrijenhoek. 1994. "DNA Primers for Amplification of Mitochondrial Cytochrome c Oxidase Subunit I from Diverse Metazoan Invertebrates." *Molecular Marine Biology and Biotechnology* 3 (5): 294–99.

Katoh, Kazutaka, and Daron M. Standley. 2013. “MAFFT Multiple Sequence Alignment Software Version 7: Improvements in Performance and Usability.” *Molecular Biology and Evolution* 30 (4): 772. https://doi.org/10.1093/molbev/mst010.

Kumar, Sudhir, Glen Stecher, Michael Li, Christina Knyaz, and Koichiro Tamura. 2018. “MEGA X: Molecular Evolutionary Genetics Analysis across Computing Platforms.” *Molecular Biology and Evolution* 35 (6): 1547–49. https://doi.org/10.1093/molbev/msy096.

Ratnasingham, Sujeevan, and Paul D. N. Hebert. 2007. “BOLD: The Barcode of Life Data System (Http://Www.Barcodinglife.Org).” *Molecular Ecology Notes* 7 (3): 355–64. https://doi.org/10.1111/j.1471-8286.2007.01678.x.

Sayers, Eric W, Evan E Bolton, J Rodney Brister, Kathi Canese, Jessica Chan, Donald C Comeau, Ryan Connor, et al. 2021. “Database Resources of the National Center for Biotechnology Information.” *Nucleic Acids Research* 50 (D1): D20–26. https://doi.org/10.1093/nar/gkab1112.

Walker, Ken. 2011. “Exoneura (Exoneura) Robusta Cockerell, 1922.” Pest and Disease Image Library (PaDIL). 2011. https://www.padil.gov.au/pollinators/pest/138693.

Walsh, P. S., D. A. Metzger, and R. Higuchi. 1991. “Chelex 100 as a Medium for Simple Extraction of DNA for PCR-Based Typing from Forensic Material.” *BioTechniques* 10 (4): 506–13.

Table S1: Collection site information, and the number of nests and bees collected from each site, in each season.

| Population | Collection_site | Season | Latitude | Longitude | Date_collected | Nests_collected | Total_adults | Females | Males |
|---|---|---|---|---|---|---|---|---|---|
| Brisbane | Fahey Road 2 | summer | -27.3297 | 152.7823 | 15/01/2022 | 16 | 86 | 68 | 18 |
| Brisbane | Fahey Road 2 | spring | -27.3297 | 152.7823 | 18/09/2022 | 16 | 98 | 81 | 17 |
| Lismore | Gwydir Highway 1 | summer | -29.629 | 152.1223 | 25/01/2022 | 19 | 120 | 105 | 15 |
| Lismore | Gwydir Highway 1 | spring | -29.629 | 152.1223 | 15/09/2022 | 6 | 15 | 15 | 0 |
| Lismore | Gwydir Highway 4 | spring | -29.5921 | 152.1871 | 15/09/2022 | 6 | 29 | 27 | 2 |
| Lismore | Gwydir Highway 6 | spring | -29.5792 | 152.1934 | 15/09/2022 | 8 | 70 | 55 | 15 |
| Lismore | Gwydir Highway 5 | spring | -29.5889 | 152.1906 | 15/09/2022 | 2 | 18 | 10 | 8 |
| Lismore | Gwydir Highway 3 | spring | -29.5959 | 152.1822 | 15/09/2022 | 5 | 18 | 18 | 0 |
| Coffs Harbour | Orama Road 1 | summer | -30.4442 | 152.6806 | 21/01/2022 | 1 | 3 | 3 | 0 |
| Coffs Harbour | Osprey Drive 1 | spring | -30.5422 | 153.0177 | 14/09/2022 | 6 | 101 | 65 | 36 |
| Coffs Harbour | Clarkes Road 1 | spring | -30.4328 | 153.0415 | 14/09/2022 | 4 | 15 | 15 | 0 |
| Laurieton | Oxley Highway 1 | summer | -31.4182 | 152.2125 | 27/01/2022 | 5 | 12 | 12 | 0 |
| Laurieton | Oxley Highway 5 | summer | -31.3927 | 152.0643 | 27/01/2022 | 4 | 7 | 7 | 0 |
| Laurieton | Oxley Highway 5 | spring | -31.3927 | 152.0643 | 10/09/2022 | 3 | 6 | 6 | 0 |
| Laurieton | Oxley Highway 2 | summer | -31.4172 | 152.1579 | 27/01/2022 | 3 | 6 | 6 | 0 |
| Laurieton | Oxley Highway 2 | spring | -31.4172 | 152.1579 | 10/09/2022 | 2 | 3 | 3 | 0 |
| Laurieton | Oxley Highway 4 | summer | -31.398 | 152.1158 | 27/01/2022 | 1 | 1 | 1 | 0 |
| Laurieton | Oxley Highway 6 | spring | -31.3898 | 152.0586 | 10/09/2022 | 13 | 63 | 54 | 9 |
| Laurieton | Oxley Highway 3 | spring | -31.402 | 152.1239 | 10/09/2022 | 2 | 23 | 19 | 4 |
| Sydney | Katoomba 1 | summer | -33.7262 | 150.3162 | 1/02/2022 | 54 | 109 | 109 | 0 |
| Sydney | Katoomba 1 | spring | -33.7262 | 150.3162 | 6/09/2022 | 20 | 59 | 55 | 4 |
| Sydney | Katoomba 1 | spring | -33.7262 | 150.3162 | 7/09/2022 | 12 | 26 | 23 | 3 |
| Batemans Bay | Durass Drive 1 | summer | -35.6608 | 150.2493 | 8/02/2022 | 6 | 45 | 31 | 14 |
| Batemans Bay | Durass Drive 1 | spring | -35.6608 | 150.2493 | 4/09/2022 | 3 | 26 | 23 | 3 |
| Batemans Bay | Wrights Road 1 | spring | -35.6583 | 150.2408 | 4/09/2022 | 10 | 127 | 96 | 31 |

| | | | | | | | | | |
|---|---|---|---|---|---|---|---|---|---|
| Baw Baw | Mt Baw Baw Tourist Road 1 | summer | -37.8488 | 146.2295 | 21/02/2022 | 29 | 38 | 38 | 0 |
| Baw Baw | Mt Baw Baw Tourist Road 1 | spring | -37.8488 | 146.2295 | 27/09/2022 | 25 | 81 | 80 | 1 |
| Wilsons Promontory | Lilly Pilly Gully Track 2 | summer | -39.0182 | 146.327 | 17/02/2022 | 5 | 9 | 9 | 0 |
| Wilsons Promontory | Lilly Pilly Gully Track 2 | spring | -39.0182 | 146.327 | 31/08/2022 | 10 | 46 | 39 | 7 |
| Wilsons Promontory | Lilly Pilly Gully Track 4 | summer | -39.0181 | 146.3305 | 17/02/2022 | 17 | 43 | 43 | 0 |
| Wilsons Promontory | Lilly Pilly Gully Track 1 | summer | -39.019 | 146.3241 | 17/02/2022 | 6 | 26 | 26 | 0 |
| Wilsons Promontory | Lilly Pilly Gully Track 3 | summer | -39.0179 | 146.3277 | 17/02/2022 | 1 | 2 | 2 | 0 |
| Wilsons Promontory | Lilly Pilly Gully Track 8 | spring | -39.0209 | 146.3297 | 31/08/2022 | 21 | 106 | 77 | 29 |

Table S2: Sample sizes of bees for each thermal tolerance assay in the field, and the number of nests that these bees came from. Note that bees from the same nest were divided between the two thermal tolerance assays as often as possible.

| Assay | Population | Season | Nests_used | Total_adults | Females | Males |
|---|---|---|---|---|---|---|
| CTmax | Brisbane | summer | 10 | 47 | 46 | 1 |
| CTmax | Brisbane | spring | 13 | 48 | 41 | 7 |
| CTmax | Lismore | summer | 17 | 61 | 51 | 10 |
| CTmax | Lismore | spring | 24 | 78 | 68 | 10 |
| CTmax | Coffs Harbour | summer | 1 | 1 | 1 | 0 |
| CTmax | Coffs Harbour | spring | 10 | 57 | 39 | 18 |
| CTmax | Laurieton | summer | 7 | 10 | 10 | 0 |
| CTmax | Laurieton | spring | 15 | 43 | 37 | 6 |
| CTmax | Sydney | summer | 45 | 68 | 68 | 0 |
| CTmax | Sydney | spring | 21 | 41 | 38 | 3 |
| CTmax | Batemans Bay | summer | 6 | 22 | 13 | 9 |
| CTmax | Batemans Bay | spring | 13 | 78 | 56 | 22 |
| CTmax | Baw Baw | summer | 22 | 23 | 23 | 0 |
| CTmax | Baw Baw | spring | 23 | 40 | 40 | 0 |
| CTmax | Wilsons Promontory | summer | 21 | 44 | 44 | 0 |
| CTmax | Wilsons Promontory | spring | 25 | 74 | 54 | 20 |
| CTmin | Brisbane | summer | 8 | 39 | 22 | 17 |
| CTmin | Brisbane | spring | 16 | 50 | 40 | 10 |
| CTmin | Lismore | summer | 18 | 59 | 54 | 5 |
| CTmin | Lismore | spring | 23 | 72 | 57 | 15 |
| CTmin | Coffs Harbour | summer | 1 | 2 | 2 | 0 |
| CTmin | Coffs Harbour | spring | 10 | 59 | 41 | 18 |
| CTmin | Laurieton | summer | 11 | 16 | 16 | 0 |
| CTmin | Laurieton | spring | 20 | 52 | 45 | 7 |
| CTmin | Sydney | summer | 32 | 41 | 41 | 0 |
| CTmin | Sydney | spring | 26 | 44 | 40 | 4 |
| CTmin | Batemans Bay | summer | 5 | 23 | 18 | 5 |
| CTmin | Batemans Bay | spring | 12 | 75 | 63 | 12 |
| CTmin | Baw Baw | summer | 13 | 15 | 15 | 0 |
| CTmin | Baw Baw | spring | 23 | 41 | 40 | 1 |
| CTmin | Wilsons Promontory | summer | 26 | 36 | 36 | 0 |
| CTmin | Wilsons Promontory | spring | 28 | 78 | 62 | 16 |

Table S3: Sample sizes for thermal tolerance assays following 24-days of adult acclimation. The table shows the number of nests that contained bees at the end of the experiment, in addition to the total number of adult female bees

| Assay | Population | Acclimation_temperature | Nests | Females |
|---|---|---|---|---|
| CTmax | Brisbane | 26 | 14 | 26 |
| CTmax | Brisbane | 21 | 10 | 15 |
| CTmax | Sydney | 26 | 14 | 28 |
| CTmax | Sydney | 21 | 15 | 22 |
| CTmax | Baw Baw | 26 | 14 | 29 |
| CTmax | Baw Baw | 21 | 14 | 30 |
| CTmin | Brisbane | 26 | 13 | 25 |
| CTmin | Brisbane | 21 | 12 | 17 |
| CTmin | Sydney | 26 | 12 | 26 |
| CTmin | Sydney | 21 | 15 | 22 |
| CTmin | Baw Baw | 26 | 12 | 27 |
| CTmin | Baw Baw | 21 | 15 | 26 |

Table S4: Field acclimation in cold tolerance, post-hoc results.

| Season | Population | contrast | estimate | SE | df | t.ratio | p.value |
|---|---|---|---|---|---|---|---|
| . | Brisbane | summer - spring | 0.39 | 0.19 | 89.24 | 2.06 | 0.070 |
| . | Lismore | summer - spring | -1.11 | 0.13 | 119.06 | -8.23 | ***1.78E-12*** |
| . | Coffs Harbour | summer - spring | -0.87 | 0.49 | 293.71 | -1.77 | 0.122 |
| . | Laurieton | summer - spring | -0.51 | 0.2 | 309.69 | -2.57 | ***0.021*** |
| . | Sydney | summer - spring | -0.41 | 0.15 | 379.3 | -2.7 | ***0.015*** |
| . | Batemans Bay | summer - spring | -0.18 | 0.19 | 90.34 | -0.97 | 0.429 |
| . | Baw Baw | summer - spring | 0.81 | 0.2 | 447.63 | 3.96 | ***2.18E-04*** |
| . | Wilsons Promontory | summer - spring | 0.51 | 0.14 | 301.81 | 3.6 | ***8.50E-04*** |
| summer | . | Brisbane - Lismore | 2.49 | 0.18 | 82.05 | 13.66 | ***2.49E-21*** |
| summer | . | Brisbane - Coffs Harbour | 2.44 | 0.5 | 290.31 | 4.84 | ***5.82E-06*** |
| summer | . | Brisbane - Laurieton | 2.23 | 0.23 | 165.84 | 9.7 | ***1.04E-16*** |
| summer | . | Brisbane - Sydney | 2.04 | 0.19 | 123.43 | 10.9 | ***1.87E-18*** |
| summer | . | Brisbane - Batemans Bay | 1.88 | 0.23 | 92.03 | 8.26 | ***6.14E-12*** |
| summer | . | Brisbane - Baw Baw | 1.61 | 0.23 | 204.9 | 6.94 | ***2.46E-10*** |
| summer | . | Brisbane - Wilsons Promontory | 1.1 | 0.19 | 122.35 | 5.77 | ***2.19E-07*** |
| summer | . | Lismore - Coffs Harbour | -0.06 | 0.49 | 324.2 | -0.12 | 0.921 |
| summer | . | Lismore - Laurieton | -0.27 | 0.2 | 262.13 | -1.37 | 0.244 |
| summer | . | Lismore - Sydney | -0.46 | 0.14 | 222.21 | -3.2 | ***0.003*** |

| | | | | | | | |
|---|---|---|---|---|---|---|---|
| summer | . | Lismore - Batemans Bay | -0.62 | 0.19 | 108.08 | -3.2 | ***0.004*** |
| summer | . | Lismore - Baw Baw | -0.88 | 0.2 | 351.58 | -4.47 | ***2.75E-05*** |
| summer | . | Lismore - Wilsons Promontory | -1.39 | 0.15 | 207.92 | -9.33 | ***2.13E-16*** |
| summer | . | Coffs Harbour - Laurieton | -0.21 | 0.51 | 344.24 | -0.41 | 0.729 |
| summer | . | Coffs Harbour - Sydney | -0.4 | 0.49 | 346.24 | -0.82 | 0.498 |
| summer | . | Coffs Harbour - Batemans Bay | -0.56 | 0.51 | 299.3 | -1.1 | 0.361 |
| summer | . | Coffs Harbour - Baw Baw | -0.83 | 0.51 | 359.09 | -1.63 | 0.156 |
| summer | . | Coffs Harbour - Wilsons Promontory | -1.33 | 0.49 | 343.05 | -2.71 | ***0.015*** |
| summer | . | Laurieton - Sydney | -0.19 | 0.2 | 393.76 | -0.95 | 0.429 |
| summer | . | Laurieton - Batemans Bay | -0.35 | 0.24 | 196.23 | -1.47 | 0.209 |
| summer | . | Laurieton - Baw Baw | -0.62 | 0.24 | 439.42 | -2.55 | ***0.021*** |
| summer | . | Laurieton - Wilsons Promontory | -1.12 | 0.2 | 371.76 | -5.51 | ***2.29E-07*** |
| summer | . | Sydney - Batemans Bay | -0.16 | 0.2 | 159.7 | -0.81 | 0.498 |
| summer | . | Sydney - Baw Baw | -0.43 | 0.2 | 493.47 | -2.11 | 0.060 |
| summer | . | Sydney - Wilsons Promontory | -0.93 | 0.15 | 415.23 | -6.04 | ***1.35E-08*** |
| summer | . | Batemans Bay - Baw Baw | -0.27 | 0.24 | 239.8 | -1.12 | 0.361 |
| summer | . | Batemans Bay - Wilsons Promontory | -0.77 | 0.2 | 156.53 | -3.84 | ***4.29E-04*** |
| summer | . | Baw Baw - Wilsons Promontory | -0.5 | 0.21 | 468.45 | -2.45 | ***0.027*** |
| spring | . | Brisbane - Lismore | 0.99 | 0.15 | 132.7 | 6.84 | ***1.21E-09*** |
| spring | . | Brisbane - Coffs Harbour | 1.17 | 0.16 | 68.24 | 7.15 | ***3.21E-09*** |
| spring | . | Brisbane - Laurieton | 1.32 | 0.15 | 152.72 | 8.69 | ***5.47E-14*** |
| spring | . | Brisbane - Sydney | 1.24 | 0.16 | 190.83 | 7.97 | ***9.97E-13*** |
| spring | . | Brisbane - Batemans Bay | 1.3 | 0.15 | 84.62 | 8.94 | ***5.81E-13*** |
| spring | . | Brisbane - Baw Baw | 2.02 | 0.16 | 188.77 | 13.02 | ***2.86E-26*** |
| spring | . | Brisbane - Wilsons Promontory | 1.23 | 0.14 | 151.67 | 8.64 | ***6.79E-14*** |
| spring | . | Lismore - Coffs Harbour | 0.18 | 0.15 | 64.48 | 1.17 | 0.345 |
| spring | . | Lismore - Laurieton | 0.33 | 0.14 | 167.74 | 2.36 | ***0.034*** |

| | | | | | | | |
|---|---|---|---|---|---|---|---|
| spring | . | Lismore - Sydney | 0.24 | 0.14 | 219.29 | 1.71 | 0.136 |
| spring | . | Lismore - Batemans Bay | 0.31 | 0.13 | 81.91 | 2.32 | ***0.039*** |
| spring | . | Lismore - Baw Baw | 1.03 | 0.14 | 216.38 | 7.21 | ***4.93E-11*** |
| spring | . | Lismore - Wilsons Promontory | 0.23 | 0.13 | 169.18 | 1.81 | 0.114 |
| spring | . | Coffs Harbour - Laurieton | 0.15 | 0.16 | 75.15 | 0.96 | 0.429 |
| spring | . | Coffs Harbour - Sydney | 0.07 | 0.16 | 90.44 | 0.41 | 0.729 |
| spring | . | Coffs Harbour - Batemans Bay | 0.13 | 0.15 | 46.85 | 0.85 | 0.495 |
| spring | . | Coffs Harbour - Baw Baw | 0.85 | 0.16 | 89.74 | 5.27 | ***2.82E-06*** |
| spring | . | Coffs Harbour - Wilsons Promontory | 0.06 | 0.15 | 68.81 | 0.37 | 0.747 |
| spring | . | Laurieton - Sydney | -0.09 | 0.15 | 246.23 | -0.57 | 0.638 |
| spring | . | Laurieton - Batemans Bay | -0.02 | 0.14 | 99.03 | -0.17 | 0.896 |
| spring | . | Laurieton - Baw Baw | 0.7 | 0.15 | 243.2 | 4.68 | ***1.28E-05*** |
| spring | . | Laurieton - Wilsons Promontory | -0.1 | 0.14 | 198.95 | -0.71 | 0.556 |
| spring | . | Sydney - Batemans Bay | 0.06 | 0.14 | 127.07 | 0.44 | 0.729 |
| spring | . | Sydney - Baw Baw | 0.78 | 0.15 | 306.4 | 5.14 | ***1.56E-06*** |
| spring | . | Sydney - Wilsons Promontory | -0.01 | 0.14 | 263.95 | -0.08 | 0.937 |
| spring | . | Batemans Bay - Baw Baw | 0.72 | 0.14 | 125.66 | 5.07 | ***4.04E-06*** |
| spring | . | Batemans Bay - Wilsons Promontory | -0.07 | 0.13 | 91.51 | -0.57 | 0.638 |
| spring | . | Baw Baw - Wilsons Promontory | -0.8 | 0.14 | 260.16 | -5.72 | ***1.10E-07*** |

Table S5: Post-hoc comparisons of heat tolerance in populations from the acclimation experiment

| contrast | estimate | SE | Df | t.ratio | p.value |
|---|---|---|---|---|---|
| Brisbane - Sydney | -0.38 | 0.15 | 76.22 | -2.52 | ***0.041*** |
| Brisbane - Baw Baw | -0.25 | 0.15 | 66.81 | -1.71 | 0.138 |
| Sydney - Baw Baw | 0.13 | 0.14 | 63.54 | 0.93 | 0.356 |

Table S6: Post-hoc comparisons of heat tolerance in the field, for the three populations used in the acclimation experiment

| contrast | Estimate | SE | df | t.ratio | p.value |
|---|---|---|---|---|---|
| Brisbane - Sydney | -0.39 | 0.14 | 74.51 | -2.85 | ***0.008*** |
| Brisbane - Baw Baw | -0.02 | 0.15 | 90.90 | -0.13 | 0.898 |

| | | | | | |
|---|---|---|---|---|---|
| Sydney - Baw Baw | 0.37 | 0.13 | 143.34 | 2.88 | ***0.008*** |

Table S7: Post-hoc comparisons of cold tolerance field acclimation, for the three populations used in the acclimation experiment.

| Season | Population | contrast | estimate | SE | df | t.ratio | p.value |
|---|---|---|---|---|---|---|---|
| . | Brisbane | summer - spring | 0.41 | 0.19 | 16.87 | 2.14 | ***0.049*** |
| . | Sydney | summer - spring | -0.4 | 0.16 | 114.6 | -2.53 | ***0.016*** |
| . | Baw Baw | summer - spring | 0.81 | 0.21 | 144.3 | 3.77 | ***3.60E-04*** |
| summer | . | Brisbane - Sydney | 2.06 | 0.19 | 25.8 | 10.77 | ***2.20E-10*** |
| summer | . | Brisbane - Baw Baw | 1.64 | 0.24 | 49.83 | 6.82 | ***2.64E-08*** |
| summer | . | Sydney - Baw Baw | -0.42 | 0.21 | 163.83 | -1.98 | ***0.049*** |
| spring | . | Brisbane - Sydney | 1.25 | 0.16 | 45.04 | 7.76 | ***2.28E-09*** |
| spring | . | Brisbane - Baw Baw | 2.03 | 0.16 | 45.04 | 12.66 | ***1.74E-15*** |
| spring | . | Sydney - Baw Baw | 0.78 | 0.16 | 85.96 | 4.92 | ***7.35E-06*** |

a)

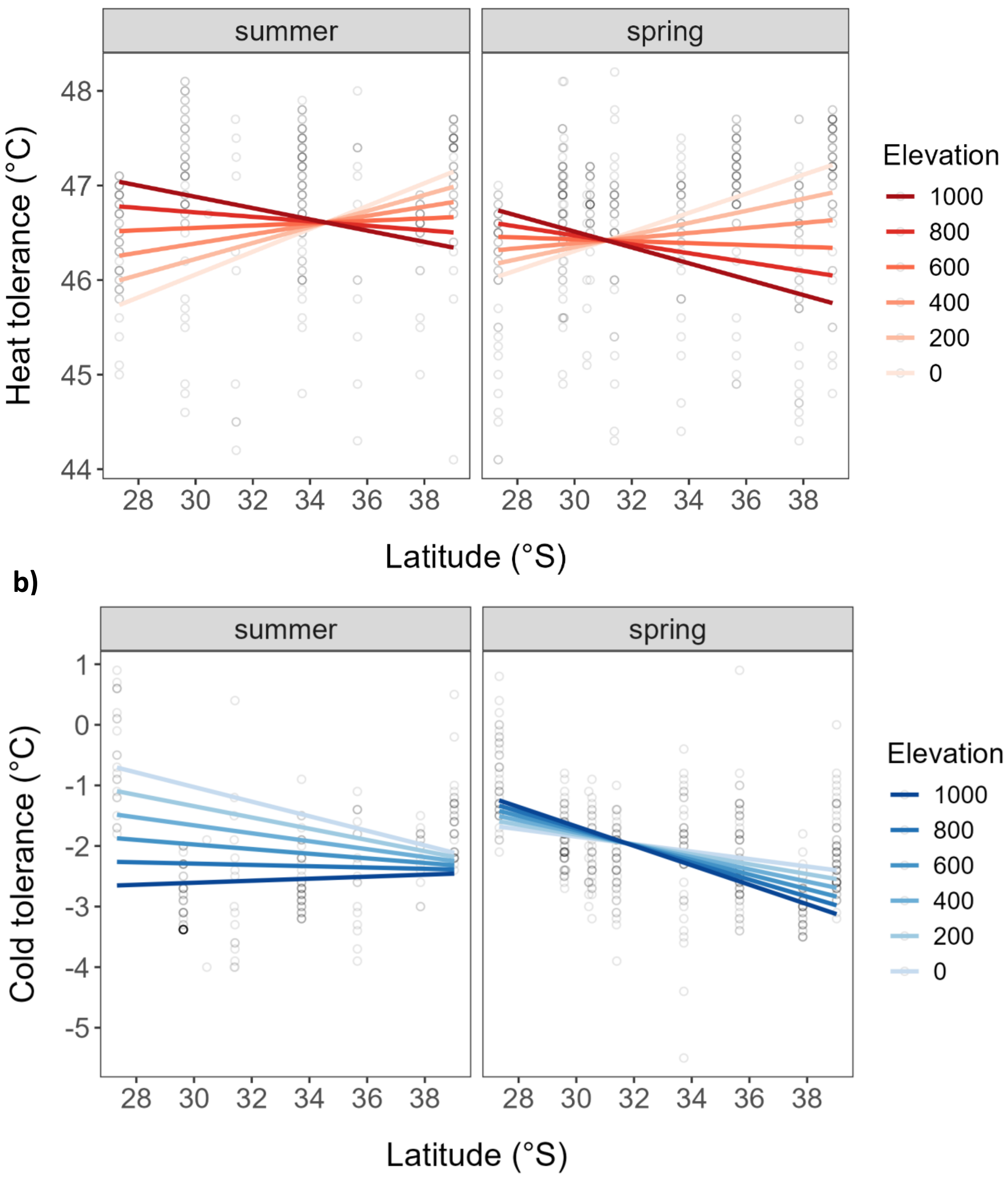


Figure S1: Female *Exoneura robusta* thermal tolerance interactions along a latitudinal cline, where trend lines show model predictions for different elevations, and points represent the raw data. a) Heat tolerance depended on the two-way interaction between latitude and elevation, and the two-way interaction between elevation and season. b) Cold tolerance depended on the three-way interaction between latitude, elevation and season.

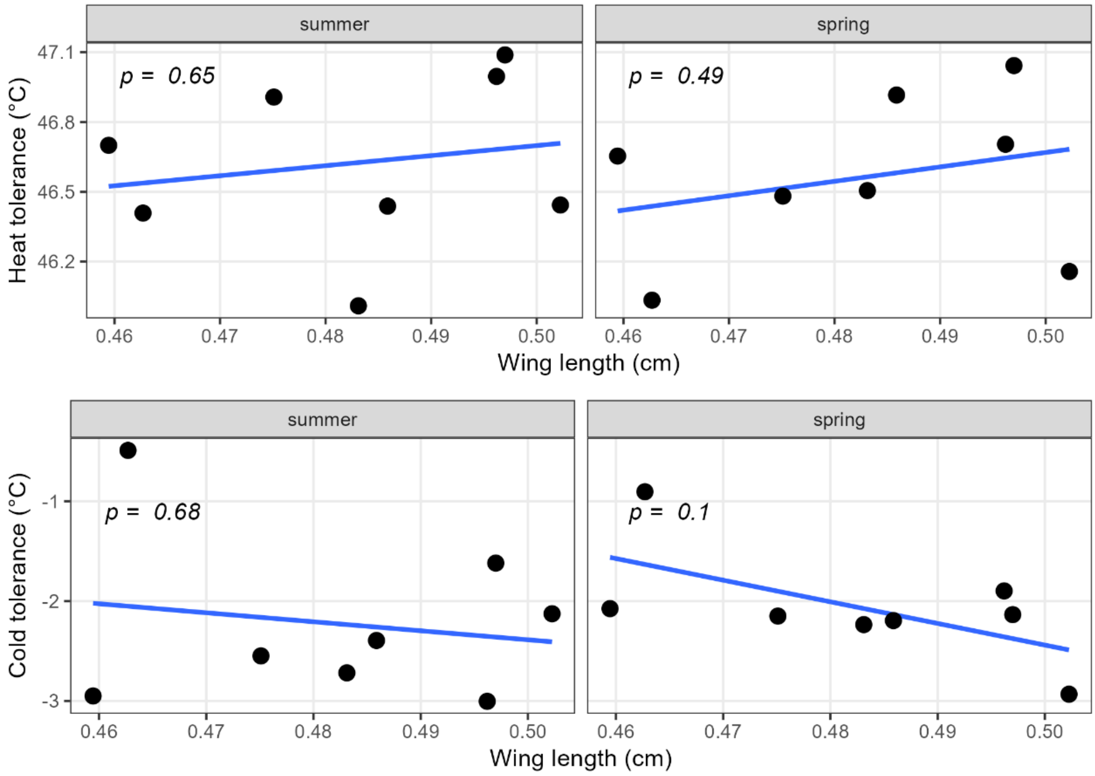


Figure S2: We found no relationship between wing length (a proxy for body size) and thermal tolerance.
